# Association of accelerometer-derived sleep characteristics with incident osteoporosis: a prospective cohort study from the UK Biobank

**DOI:** 10.3389/fendo.2026.1818773

**Published:** 2026-08-05

**Authors:** Zhenyu Hu, Guosheng Wang, Xiaoyi Kong, Jingfang Jia, Mengjing Zhao, Jinmei Deng, Yang Cao, Guoqi Cai, Faming Pan

**Affiliations:** 1 Health Management Center, The Fourth Affiliated Hospital of Anhui Medical University (Chaohu Hospital of Anhui Medical University), Hefei, Anhui, China; 2 Department of Epidemiology and Biostatistics, School of Public Health, Anhui Medical University, Hefei, Anhui, China; 3 The Key Laboratory of Major Autoimmune Diseases, Anhui Medical University, Hefei, Anhui, China

**Keywords:** circadian rhythm, osteoporosis, relative amplitude, sleep duration, sleep efficiency, UK Biobank

## Abstract

**Background:**

Osteoporosis (OP) is a common metabolic bone disease whose onset and progression are influenced by sleep patterns. However, previous related studies have mostly relied on self-reported sleep data and have often been confined to a single dimension such as sleep duration. Therefore, this study aimed to evaluate the associations between multidimensional, objective sleep characteristics and OP.

**Methods:**

This study was based on the UK Biobank and included 72,211 participants. Five objective sleep metrics were derived from 7 consecutive days of wrist accelerometer data: sleep duration, sleep efficiency, sleep regularity index, sleep midpoint, and relative amplitude. Incident OP events were identified through linkage with electronic medical records. Multivariable Cox proportional hazards models were used to examine the associations between objective sleep metrics and OP. Restricted cubic splines (RCS) were applied to test potential nonlinear relationships. Interaction analyses and sex-stratified subgroup analyses were also conducted based on the above results.

**Results:**

Average sleep duration (HR = 0.91, 95% CI: 0.87–0.95; *P* < 0.001), sleep efficiency (HR = 0.92, 95% CI: 0.88–0.97; *P* = 0.001), and relative amplitude (HR = 0.80, 95% CI: 0.76–0.85; *P* < 0.001) were all associated with a reduced risk of OP. RCS analyses showed no significant nonlinear effects (all *P*_nonlinear_ > 0.05). Interaction analysis suggested a significant negative interaction between relative amplitude and sleep duration (HR 0.96, 95% CI 0.93–0.99; *P* = 0.012). In sex-stratified subgroup analyses, the pattern of associations in women was consistent with the main analysis, whereas in men only sleep duration and relative amplitude were significantly associated with lower OP risk (all *P* < 0.05).

**Conclusions:**

Longer objective sleep duration, higher sleep efficiency, and stronger relative amplitude were independently associated with a lower risk of OP. The association between sleep duration and OP also varied by relative amplitude. These findings suggest that objective sleep characteristics and behavioral rest-activity rhythmicity may be relevant to OP risk, although further mechanistic and interventional studies are needed to clarify causality.

## Introduction

Osteoporosis (OP) is one of the most common diseases among older adults. More than 200 million people worldwide are affected. Characterized primarily by reduced bone mass and deterioration of bone microarchitecture, OP markedly increases fracture risk and imposes a substantial disease burden (1, 2).

OP essentially results from disruption of the balance between bone resorption and bone formation in bone metabolism. Beyond traditional factors such as genetics and sex hormones, bone metabolism itself shows pronounced rhythmic features: metabolic processes are precisely regulated by internal clocks within osteoblasts and osteoclasts (3). This suggests a potential influence of sleep rhythms on skeletal health, and disruption of sleep rhythms may be a potential risk factor for OP.

Many previous studies have examined the association between sleep and OP (or bone mineral density). A meta-analysis reported that both excessively long and short sleep duration were associated with decreased bone mineral density (4), and good sleep quality was considered to be associated with a lower risk of OP (5). However, most existing studies assess sleep primarily using self-reported measures, making them vulnerable to recall bias, and they largely focus on single dimensions such as sleep duration or sleep quality. Given the physiological basis of the skeletal biological clock, sleep quantity or quality alone cannot fully reflect the stability of sleep rhythms. It is therefore necessary to introduce more dimensions of sleep characteristics for systematic analysis. Accordingly, leveraging the accelerometer sub-cohort of the UK Biobank (UKB), this study systematically investigated the associations of objective sleep duration, sleep efficiency, sleep regularity index, sleep midpoint, and relative amplitude derived from accelerometer data with OP risk, with the aim of providing more comprehensive evidence for future research on sleep-related behavioral patterns and skeletal health.

## Methods

### Participants

This study was conducted within the UKB cohort (project ID 96569). UKB is a large prospective cohort study involving over 500,000 participants in the United Kingdom, initiated in 2006. Between 2013 and 2015, approximately 100,000 participants were invited to wear a wrist-mounted triaxial accelerometer (Axivity AX3) continuously for one week to objectively assess activity and sleep (6).

After identifying all participants who wore accelerometers, we excluded those with poor data quality (uncalibrated data or fewer than 7 days of sleep data) and those with OP prior to accelerometer wear. Ultimately, 72,211 participants were included (see **Supplementary Figure 1**). UKB has received approval from the North West Multi-center Research Ethics Committee (REC reference: 21/NW/0157), and all participants provided written informed consent (7).

### Exposures

We used the GGIR package to analyze raw accelerometer data (8) and extracted five objective sleep metrics to reflect participants’ sleep patterns and rhythms: sleep duration, sleep efficiency, sleep regularity index, sleep midpoint, and relative amplitude. Details of accelerometer processing and derivation of sleep metrics are provided in **Supplementary Method 1**. To remove the influence of scale, all exposure variables were Z-score standardized before inclusion in models. To facilitate clinical interpretation, the distributions of sleep metrics in their original units, including mean, standard deviation, and range, were also summarized.

### Outcomes

The outcome of this study was incident OP during follow-up. The date of starting accelerometer wear was defined as the start of follow-up, and incident OP was defined as the first OP record occurring after this date. Follow-up continued until OP occurrence, death, or the administrative censoring date (March 31, 2023 for England; August 31, 2022 for Scotland; May 31, 2022 for Wales). OP events were identified from primary care records, hospital inpatient databases, and self-reported medical history. The corresponding ICD-10 codes were M80 (OP with pathological fracture) and M81 (OP without pathological fracture). Participants with an OP record before accelerometer wear were excluded to reduce prevalent-case contamination.

### Covariates

Based on previous studies related to OP or sleep (8–12) and a conceptual directed acyclic graph (DAG; **Supplementary Figure 3**), the following covariates were included in the primary adjusted model to account for potential confounding and baseline health-related differences. Demographic variables: age, sex, ethnicity, education level, Townsend deprivation index (TDI), and body mass index (BMI). Lifestyle variables: smoking, alcohol consumption, shift work, and moderate-to-vigorous physical activity (MVPA; minutes/week). Health-related variables: diabetes, hypertension, cardiovascular disease, sleep disorders, depression, chronic pain, and medications affecting bone metabolism (systemic glucocorticoids, bisphosphonates, oral contraceptives). MVPA, sleep disorders, depression, and chronic pain may have dual roles as potential confounders and mediator-like variables; therefore, they were included in the primary model and excluded in sensitivity analyses to assess possible overadjustment. Missing covariates were imputed using multiple imputation by chained equations (13), with missingness and imputation methods summarized in **Supplementary Table 1**.

### Statistical analysis

Cox proportional hazards models were used, with stepwise adjustment of covariates, yielding three models: Model 1 adjusted for demographic variables only; Model 2 further adjusted for lifestyle variables; Model 3 adjusted for all covariates. The proportional hazards assumption was assessed using Schoenfeld residuals. To explore potential nonlinear effects, restricted cubic spline (RCS) analyses with 4 knots were performed and exposure–response curves were plotted (14). Given the marked sex differences in OP incidence, sex-stratified subgroup analyses were conducted after full adjustment. For the female subgroup, menopausal status and hormone replacement therapy (HRT) were additionally adjusted. Effect modification by sex was further assessed by including interaction terms between sex and each sleep metric. In addition, multiplicative interaction analyses were performed for sleep duration × sleep efficiency, sleep duration × relative amplitude, and sleep efficiency × relative amplitude. For statistically significant interactions, joint exposure analyses were performed by categorizing the corresponding two metrics into tertiles and visualized using heatmaps, with the lowest tertile for both metrics as the reference group.

In addition, several sensitivity analyses were conducted to ensure robustness: (1) delaying the start of follow-up by 1, 2, and 3 years and reanalyzing after excluding participants who developed incident OP during the corresponding lag period, to reduce reverse causation; (2) resetting the censoring date to December 31, 2019, to exclude the impact of the COVID-19 pandemic; and (3) treating death as a competing event and reanalyzing using Fine–Gray subdistribution hazard models; (4) conducting an analysis restricted to postmenopausal women; (5) excluding potential mediator-like variables, including MVPA, depression, chronic pain, and sleep disorders, to assess possible overadjustment.

All analyses were performed in R (version 4.4.3). A two-sided P < 0.05 was considered statistically significant.

## Results

### Baseline characteristics

Among the 72,211 participants included, 1,719 OP events occurred during follow-up. Compared with those without OP, participants who developed OP had a higher proportion of women, were older, had higher TDI, and had higher prevalences of cardiovascular disease, hypertension, depression, chronic pain, and use of glucocorticoids and bisphosphonates, while having a lower BMI. Education level, smoking, alcohol consumption, and shift work also differed significantly between groups (see **Table 1**). Spearman correlation analyses were also conducted among objective sleep characteristics (**Supplementary Figure 2**). All characteristics were significantly correlated, but correlations were moderate or weak, with the maximum absolute correlation coefficient not exceeding 0.46.

**Table 1:** Baseline characteristics of the study population according to incident osteoporosis status.

Characteristic	Overall N = 72211^1^	No OP N = 70492^1^	OP N = 1719^1^	*P* value^2^	SMD
Age	62.52 (7.78)	62.43 (7.79)	66.29 (6.35)	<0.001	0.543
Sex				<0.001	0.666
Female	39,814 (55.1%)	38,376 (54.4%)	1,438 (83.7%)		
Male	32,397 (44.9%)	32,116 (45.6%)	281 (16.3%)		
BMI	26.70 (4.49)	26.73 (4.49)	25.32 (4.43)	<0.001	0.317
Ethnicity				0.088	0.046
White	70,099 (97.1%)	68,418 (97.1%)	1,681 (97.8%)		
Other	2,112 (2.9%)	2,074 (2.9%)	38 (2.2%)		
TDI	-1.75 (2.80)	-1.76 (2.80)	-1.59 (2.82)	0.018	0.058
Education level				<0.001	0.19
ISCED 1	6,090 (8.4%)	5,905 (8.4%)	185 (10.8%)		
ISCED 2	10,634 (14.7%)	10,314 (14.6%)	320 (18.6%)		
ISCED 3	4,493 (6.2%)	4,380 (6.2%)	113 (6.6%)		
ISCED 4	9,108 (12.6%)	8,850 (12.6%)	258 (15.0%)		
ISCED 5	41,886 (58.0%)	41,043 (58.2%)	843 (49.0%)		
Smoking				0.003	0.083
Never	41,336 (57.2%)	40,420 (57.3%)	916 (53.3%)		
Previous	26,081 (36.1%)	25,410 (36.0%)	671 (39.0%)		
Current	4,794 (6.6%)	4,662 (6.6%)	132 (7.7%)		
Alcohol				<0.001	0.121
Never	2,065 (2.9%)	2,001 (2.8%)	64 (3.7%)		
Previous	1,935 (2.7%)	1,855 (2.6%)	80 (4.7%)		
Current	68,211 (94.5%)	66,636 (94.5%)	1,575 (91.6%)		
MVPA	113.30 (49.24)	113.52 (49.21)	104.25 (49.92)	<0.001	0.187
Diabetes				0.3	0.025
No	69,050 (95.6%)	67,415 (95.6%)	1,635 (95.1%)		
Yes	3,161 (4.4%)	3,077 (4.4%)	84 (4.9%)		
CVD				0.002	0.071
No	67,061 (92.9%)	65,497 (92.9%)	1,564 (91.0%)		
Yes	5,150 (7.1%)	4,995 (7.1%)	155 (9.0%)		
Hypertension				0.046	0.049
No	52,426 (72.6%)	51,215 (72.7%)	1,211 (70.4%)		
Yes	19,785 (27.4%)	19,277 (27.3%)	508 (29.6%)		
Sleep Disorder				0.090	0.041
No	70,973 (98.3%)	69,293 (98.3%)	1,680 (97.7%)		
Yes	1,238 (1.7%)	1,199 (1.7%)	39 (2.3%)		
Depression				<0.001	0.117
No	64,803 (89.7%)	63,324 (89.8%)	1,479 (86.0%)		
Yes	7,408 (10.3%)	7,168 (10.2%)	240 (14.0%)		
Shift work				<0.001	0.349
no shift work	43,397 (60.1%)	42,629 (60.5%)	768 (44.7%)		
day shift work	1,970 (2.7%)	1,937 (2.7%)	33 (1.9%)		
night shift work	1,045 (1.4%)	1,027 (1.5%)	18 (1.0%)		
Not working or Retired	25,799 (35.7%)	24,899 (35.3%)	900 (52.4%)		
Pain Chronic				<0.001	0.132
No	46,236 (64.0%)	45,243 (64.2%)	993 (57.8%)		
Yes	25,975 (36.0%)	25,249 (35.8%)	726 (42.2%)		
Glucocorticoid				<0.001	0.121
No	71,841 (99.5%)	70,152 (99.5%)	1,689 (98.3%)		
Yes	370 (0.5%)	340 (0.5%)	30 (1.7%)		
Bisphosphonate				<0.001	0.184
No	71,894 (99.6%)	70,220 (99.6%)	1,674 (97.4%)		
Yes	317 (0.4%)	272 (0.4%)	45 (2.6%)		
Oral Contraceptive				0.7	0.016
No	72,127 (99.9%)	70,411 (99.9%)	1,716 (99.8%)		
Yes	84 (0.1%)	81 (0.1%)	3 (0.2%)		
Average sleep duration	6.46 (0.99)	6.47 (0.98)	6.42 (1.01)	0.058	0.047
Sleep efficiency	88.82 (4.77)	88.82 (4.78)	88.77 (4.69)	0.672	0.01
Sleep regularity index	60.61 (11.91)	60.59 (11.91)	61.57 (11.66)	<0.001	0.084
Sleep midpoint	23.89 (1.06)	23.89 (1.06)	23.96 (1.08)	0.006	0.068
Relative amplitude	0.90 (0.04)	0.90 (0.04)	0.89 (0.04)	<0.001	0.191

^1^ Mean (SD); n (%).

^2^ Pearson’s Chi-squared test; Welch Two Sample t-test.

SMD, Standardized Mean Difference; OP, Osteoporosis; BMI, Body Mass Index; TDI, Townsend Deprivation Index; ISCED, International Standard Classification of Education; MVPA, Moderate-to-Vigorous Physical Activity; CVD, Cardiovascular Disease.

### Objective sleep characteristics and OP

**Figure 1:**
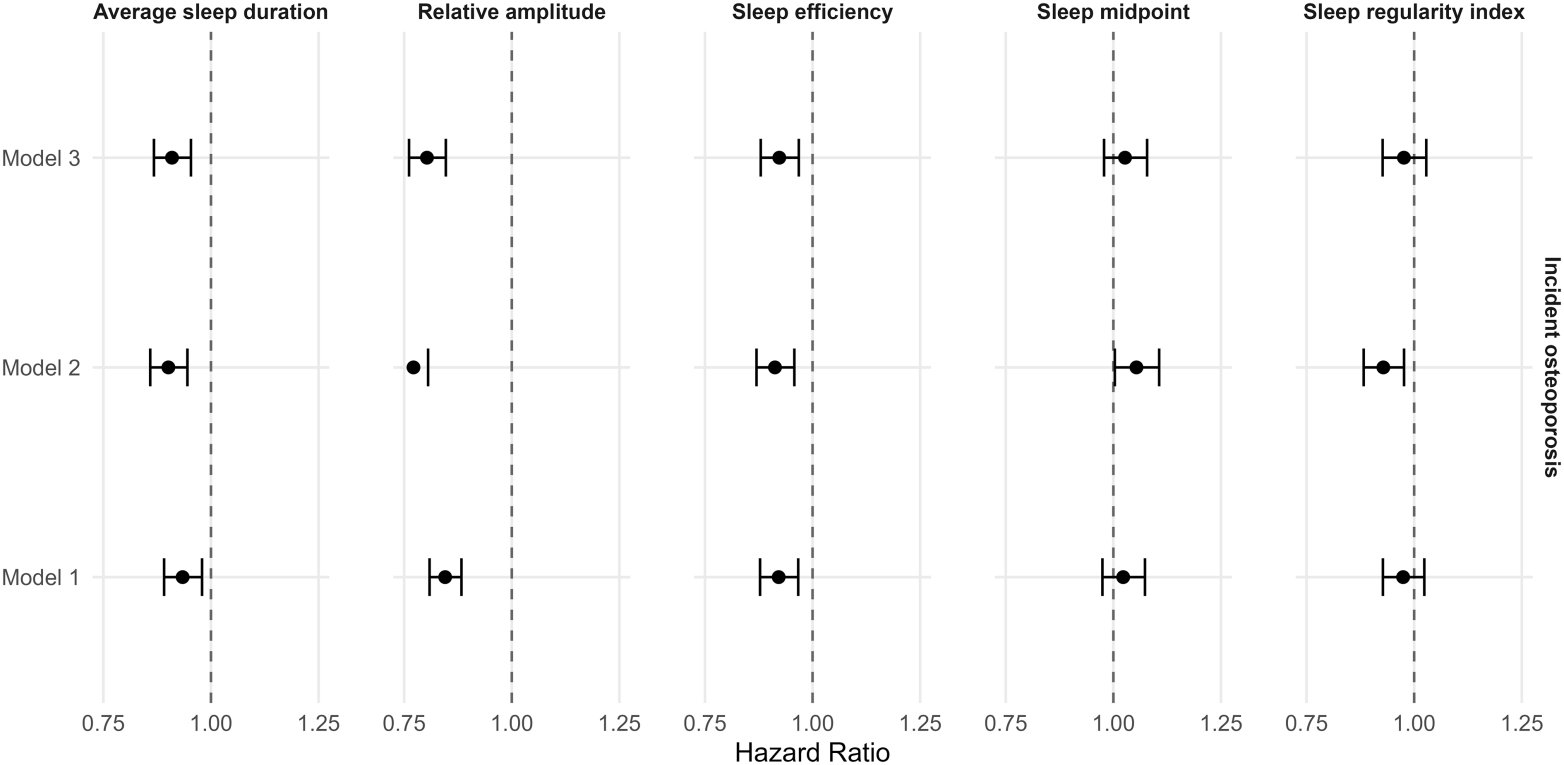
Forest plot of the associations between objective sleep characteristics and the risk of overall and osteoporosis.

As shown in **Figure 1** and **Table 2**, results from Models 1, 2, and 3 were highly consistent. Across all three models, sleep duration, sleep efficiency, and relative amplitude were significantly associated with a lower risk of OP (all *P* < 0.05). In the fully adjusted model (Model 3), each 1-SD increase in sleep duration, sleep efficiency, and relative amplitude was associated with a 9% (HR 0.91, 95% CI 0.87–0.95; *P* < 0.001), 8% (HR 0.92, 95% CI 0.88–0.97; *P* = 0.001), and 20% (HR 0.80, 95% CI 0.76–0.85; *P* < 0.001) reduction in OP risk, respectively. Sleep regularity index and sleep midpoint were significant only in Model 2 (HR 0.93, 95% CI 0.88–0.98; P = 0.004; HR 1.05, 95% CI 1.00–1.11, *P* = 0.036). No evidence of violation of the proportional hazards assumption was observed based on scaled Schoenfeld residuals (**Supplementary Table 2**). The distributions of the sleep metrics in their original units are shown in **Supplementary Table 3**. Because all sleep metrics were standardized before modeling, the HRs correspond to associations per 1-SD increase. 1 SD corresponded to approximately 0.99 hours of sleep duration, 4.8 percentage points of sleep efficiency, 11.9 units of sleep regularity index, 1.06 hours of sleep midpoint, and 0.038 units of relative amplitude.

**Table 2:** Associations of objective sleep characteristics with the risk of incident osteoporosis.

Characteristic	HR	95% CI	*P*
Model 1			
Average sleep duration	0.93	0.89-0.98	0.005
Sleep efficiency	0.92	0.88-0.97	<0.001
Sleep regularity index	0.97	0.93-1.02	0.302
Sleep midpoint	1.02	0.97-1.07	0.360
Relative amplitude	0.85	0.81-0.88	<0.001
Model 2			
Average sleep duration	0.90	0.86-0.95	<0.001
Sleep efficiency	0.91	0.87-0.96	<0.001
Sleep regularity index	0.93	0.88-0.98	0.004
Sleep midpoint	1.05	1.00-1.11	0.036
Relative amplitude	0.77	0.74-0.81	<0.001
Model 3			
Average sleep duration	0.91	0.87-0.95	<0.001
Sleep efficiency	0.92	0.88-0.97	0.001
Sleep regularity index	0.98	0.93-1.03	0.362
Sleep midpoint	1.03	0.98-1.08	0.281
Relative amplitude	0.80	0.76-0.85	<0.001

HR, Hazard Ratio; CI, Confidence Interval.

### Restricted cubic spline analysis

**Figure 2:**
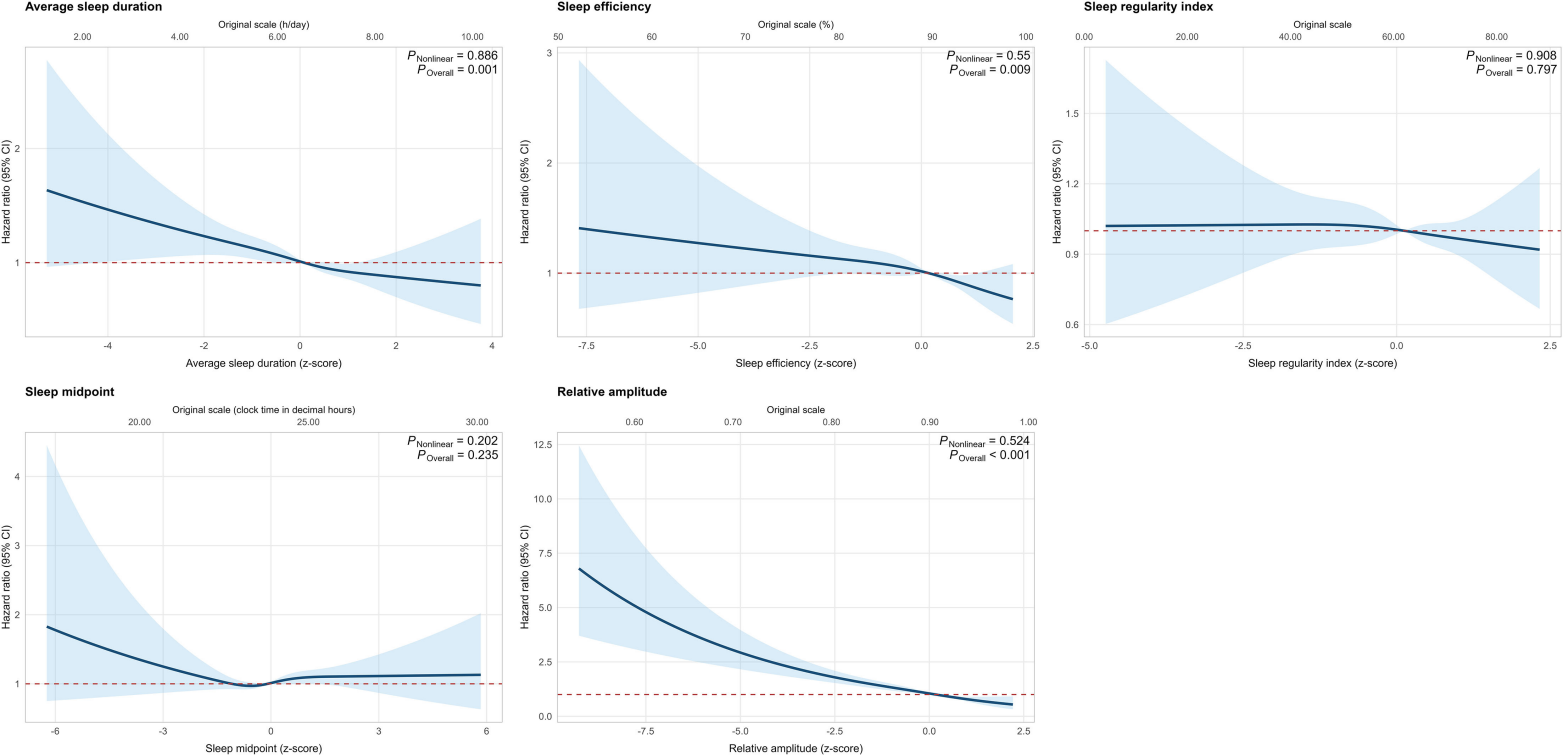
Restricted cubic spline analyses of the non-linear exposure-response relationships between objective sleep characteristics and the risk of osteoporosis.

**Figure 2** presents the exposure–response association curves between the five objective sleep characteristics and OP. However, neither sleep characteristics significant in the main analysis nor those nonsignificant showed evidence of a significant nonlinear association (all *P*_nonlinear_ > 0.05).

### Interaction analysis

**Supplementary Table 4** presents results of interaction analyses. No significant interaction effects were observed between sex and sleep duration, sleep efficiency, sleep regularity index, sleep midpoint, or relative amplitude (all P > 0.05). Neither sleep duration × sleep efficiency nor sleep efficiency × relative amplitude showed significant interaction effects (all P > 0.05), whereas sleep duration × relative amplitude showed a significant negative interaction (HR 0.96, 95% CI 0.93–0.99; *P* = 0.012).

Because the interaction between sleep duration and relative amplitude was statistically significant, we further examined their joint associations using tertile-based categories (**Supplementary Figure 4**). The heatmap showed a generally lower OP risk among participants with higher relative amplitude and longer sleep duration, supporting the presence of statistical effect modification.

### Subgroup analysis

As shown in **Supplementary Table 5**, the pattern of associations in women was highly consistent with the main analysis: sleep duration, sleep efficiency, and relative amplitude remained significantly associated with lower OP risk (HR 0.92, 95% CI 0.88–0.97; *P* = 0.002; HR 0.93, 95% CI 0.88–0.97; *P* = 0.002; HR 0.83, 95% CI 0.79–0.88; *P* < 0.001). In men, sleep efficiency was no longer significant (*P* = 0.169), while the associations for sleep duration and relative amplitude remained robust (HR 0.86, 95% CI 0.78–0.96; *P* = 0.008; HR 0.77, 95% CI 0.69–0.85; *P* < 0.001).

### Sensitivity analysis

Whether delaying the start of follow-up, using Fine–Gray subdistribution hazard models, setting the follow-up end date to December 31, 2019, or restricting the analysis to postmenopausal women, the pattern of associations between objective sleep characteristics and OP remained highly consistent with the main analysis (**Supplementary Tables 6**–**S9**). Average sleep duration, sleep efficiency, and relative amplitude remained significantly associated with a lower risk of OP (all *P* < 0.05), whereas sleep regularity index and sleep midpoint similarly showed no significant effects (all *P* > 0.05). In the sensitivity analysis excluding potential mediator-like variables, the associations for sleep duration, sleep efficiency, and relative amplitude were generally similar to those observed in the fully adjusted model. Sleep regularity index and sleep midpoint showed statistically significant associations after excluding these variables, whereas their associations were weaker and nonsignificant in the fully adjusted model, suggesting that these estimates were more sensitive to adjustment for potential mediator-like factors (**Supplementary Table 10**).

## Discussion

Using a large prospective cohort, this study systematically evaluated, for the first time, the associations between multiple objective sleep characteristics and OP. After relatively comprehensive adjustment for potential confounders, average sleep duration, sleep efficiency, and relative amplitude were all associated with a lower risk of OP, whereas no significant effects were observed for sleep midpoint or sleep regularity index. In sex-stratified subgroup analyses, the pattern of associations in women was consistent with the main analysis, while in men only sleep duration and relative amplitude were significantly associated with lower OP risk. Restricted cubic spline analyses found no evidence of significant nonlinear effects, and interaction analyses suggested a significant negative interaction between relative amplitude and sleep duration.

Most previous studies have focused on self-reported sleep duration and quality, generally indicating that shorter sleep duration or poorer sleep quality is associated with higher OP risk, consistent with our findings in terms of effect direction (4, 5). However, earlier studies also suggested that longer self-reported sleep duration is associated with increased OP risk, showing a U-shaped nonlinear relationship (4), which was not observed in our study. One possible explanation is that self-reported sleep more likely reflects time in bed rather than actual physiological sleep duration; longer time in bed itself is associated with reduced physical activity, an independent risk factor for OP (15, 16). Moreover, potential confounders such as chronic pain and depression may prolong time in bed relative to actual sleep duration (17, 18). Objective sleep duration estimated from accelerometer data more accurately reflects physiological sleep duration, which may explain the absence of the previously reported U-shaped pattern.

Sleep regularity index and sleep midpoint were not significantly associated with OP after full adjustment, despite associations in Model 2 and in the sensitivity analysis excluding potential mediator-like variables. This suggests that these two metrics may be more sensitive to adjustment for behavioral and health-related factors, including MVPA, depression, chronic pain, and sleep disorders. Their associations with OP may therefore be partly explained or mediated by these factors, whereas sleep duration, sleep efficiency, and relative amplitude showed more stable associations across models.

Consistent with prior research, relative amplitude showed the strongest association with OP risk in our study (19). Notably, the interaction analysis further revealed a negative interaction between relative amplitude and sleep duration, suggesting that the association between sleep duration and OP may differ according to the level of behavioral rest-activity rhythmicity. Given the strong sex-specific nature of OP risk, particularly the dominant role of menopause and estrogen deficiency in women, we further conducted sex-stratified analyses and formally tested effect modification by sex. The associations observed in women were largely consistent with the main findings, and these associations remained robust after additional adjustment for menopausal status and hormone replacement therapy. Moreover, sensitivity analyses restricted to postmenopausal women yielded similar results, suggesting that the observed associations between objective sleep characteristics and OP were unlikely to be fully explained by confounding from menopausal status (20).

In contrast, the associations in men should be interpreted with caution. Although sleep duration and relative amplitude remained significantly associated with lower OP risk among men, sleep efficiency was no longer statistically significant. However, formal interaction tests did not support statistically significant effect modification by sex for any sleep metric. Therefore, the apparent sex differences in subgroup-specific estimates may reflect differences in statistical power rather than true biological heterogeneity. In particular, only 281 incident OP cases occurred among men, compared with 1,438 among women, which may have limited the precision of male-specific estimates. Future studies with larger numbers of male OP cases and more detailed assessment of male-specific risk factors, including androgen status, are warranted.

Previous studies have confirmed that bone metabolism exhibits pronounced circadian rhythmicity, with the activities of osteoblasts and osteoclasts—key to maintaining bone metabolic balance—regulated by intrinsic biological clocks (21, 22). Although relative amplitude in this study was an accelerometer-derived rest-activity rhythm metric rather than a direct measure of endogenous circadian function, a higher relative amplitude reflects a clearer contrast between daytime activity and nocturnal rest and suggests a more consolidated rest-activity cycle. This behavioral rhythmicity may act as an external timing signal, influence peripheral clocks in bone tissue, and thereby affect the balance of bone metabolism, providing a possible biological context for the association between higher relative amplitude and lower OP risk (23). Meanwhile, shorter sleep duration and lower sleep efficiency may activate the hypothalamic-pituitary-adrenal axis, elevate cortisol levels, and alter its circadian rhythm (24, 25), thereby promoting bone resorption, reducing bone formation, and increasing OP risk—providing a plausible explanation for the negative interaction between sleep duration and relative amplitude. In addition, shorter sleep duration and lower sleep efficiency are associated with low-grade inflammation (26), and inflammatory cytokines can enhance osteoclast activity, disrupt bone metabolic balance, and increase OP risk. However, the specific mechanisms require further investigation.

The main strengths of this study include the large prospective cohort design, extensive adjustment for confounders, and systematic evaluation of objective sleep metrics in relation to OP risk. Multiple sensitivity analyses were highly consistent with the main findings, supporting robustness. Nevertheless, this study has limitations. First, as an observational study, residual confounding, reverse causation, and potential overadjustment cannot be fully excluded, despite the use of a conceptual DAG and multiple sensitivity analyses. Second, although the 1-, 2-, and 3-year lag analyses showed results consistent with the main findings, reverse causation cannot be fully excluded because osteoporosis develops gradually before clinical diagnosis. Undiagnosed low bone mineral density or early symptoms may have affected sleep and rest-activity patterns before OP was recorded. In addition, sleep characteristics were assessed over only one week and may not fully capture long-term sleep habits or temporal changes, which could have led to exposure misclassification and attenuation of the observed associations. Third, OP was identified from linked medical records and self-reported history rather than systematic bone mineral density testing; therefore, participants with undiagnosed low bone mineral density or clinically unrecorded OP may have been missed, leading to potential outcome misclassification and possibly attenuating the observed associations. Finally, participants in the UK Biobank were predominantly White British and generally healthier than the general population, which may limit generalizability. Because sleep patterns, rest-activity rhythms, osteoporosis risk, and diagnostic practices may differ across racial/ethnic groups and geographic regions, replication in more diverse populations is needed.

## Conclusion

Based on a large prospective cohort, this study found that longer objective sleep duration, higher sleep efficiency, and stronger relative amplitude were significantly associated with a lower risk of OP. The association between sleep duration and OP also varied by relative amplitude. These findings suggest that objective sleep characteristics and behavioral rest-activity rhythmicity may be relevant to OP risk, although further mechanistic and interventional studies are needed to clarify causality.

## Data Availability

The data that support the findings of this study are available from the UK Biobank resource upon application. Requests to access these datasets should be directed to https://www.ukbiobank.ac.uk.
