# Supplementary material for: Association of accelerometer-derived sleep characteristics with incident osteoporosis: a prospective cohort study from the UK Biobank
Source: Front Endocrinol (Lausanne). 2026 Aug 5;17:1818773. doi: 10.3389/fendo.2026.1818773 (PMC13485521; doi:10.3389/fendo.2026.1818773)
Supplement: Supplementary file 1 [file SupplementaryFile1.docx]

**Supplementary materials**

**Figure S1 Flowchart of participant selection**

**Figure S2 Spearman correlation matrix of objective sleep characteristics**

**Figure S3 Directed acyclic graph-based framework for covariate selection**

**Figure S4 Joint categories of sleep duration and relative amplitude in relation to incident osteoporosis risk**

**Table S1** **The methods employed for handling missing data**

| **Variables** | **Missing, n** | **Missing, %** | **Imputation method** |
| --- | --- | --- | --- |
| Age | 0 | 0.0 |  |
| Sex | 0 | 0.0 |  |
| Ethnicity | 239 | 0.3 | LogReg |
| Education level | 740 | 1.0 | PolyReg |
| Townsend Deprivation Index | 79 | 0.1 | PMM |
| BMI | 143 | 0.2 | PMM |
| Smoking | 194 | 0.3 | PolyReg |
| Alcohol consumption | 67 | 0.1 | PolyReg |
| Shift work | 94 | 0.1 | PolyReg |
| MVPA | 0 | 0.0 |  |
| Diabetes | 0 | 0.0 |  |
| Hypertension | 0 | 0.0 |  |
| Cardiovascular disease | 0 | 0.0 |  |
| Sleep disorders | 0 | 0.0 |  |
| Depression | 0 | 0.0 |  |
| Chronic pain | 93 | 0.1 | LogReg |
| Glucocorticoids | 0 | 0.0 |  |
| Bisphosphonates | 0 | 0.0 |  |
| Oral contraceptives | 0 | 0.0 |  |

BMI, body mass index; MVPA, moderate-to-vigorous physical activity; PMM, predictive mean matching; PolyReg, polytomous regression; LogReg, logistic regression.

**Table S2 Schoenfeld residual-based test of the proportional hazards assumption**

| **Characteristics** | **Exposure *P* value** | **Global *P* value** |
| --- | --- | --- |
| Average sleep duration |  |  |
| Model 1 | 0.183 | 0.495 |
| Model 2 | 0.184 | 0.382 |
| Model 3 | 0.218 | 0.467 |
| Sleep efficiency |  |  |
| Model 1 | 0.545 | 0.668 |
| Model 2 | 0.548 | 0.541 |
| Model 3 | 0.586 | 0.594 |
| Sleep regularity index |  |  |
| Model 1 | 0.150 | 0.439 |
| Model 2 | 0.150 | 0.322 |
| Model 3 | 0.159 | 0.453 |
| Sleep midpoint |  |  |
| Model 1 | 0.938 | 0.751 |
| Model 2 | 0.956 | 0.599 |
| Model 3 | 0.985 | 0.635 |
| Relative amplitude |  |  |
| Model 1 | 0.452 | 0.609 |
| Model 2 | 0.580 | 0.465 |
| Model 3 | 0.574 | 0.598 |

**Table S3 Distribution of accelerometer-derived sleep characteristics in original units**

| **Characteristics** | **Mean** | **SD** | **Median** | **IQR** | **Range** |
| --- | --- | --- | --- | --- | --- |
| Average sleep duration, h/day | 6.46 | 0.99 | 6.54 | 5.89~7.13 | 0.59~11.47 |
| Sleep efficiency, % | 88.80 | 4.80 | 89.60 | 86.50~92.10 | 35.70~99.30 |
| Sleep regularity index | 60.61 | 11.91 | 62.06 | 53.68~69.09 | -9.58~91.56 |
| Sleep midpoint, clock time in decimal hours | 23.89 | 1.06 | 23.79 | 23.22~24.46 | 12.49~31.26 |
| Relative amplitude | 0.90 | 0.04 | 0.91 | 0.88~0.92 | 0.33~0.99 |

IQR, interquartile range; SD, standard deviation.

**Table S4 Interaction analysis**

| **Characteristics** | **HR** | **95% CI** | ***P*** |
| --- | --- | --- | --- |
| Sleep duration×Sex | 0.92 | 0.82~1.04 | 0.285 |
| Sleep efficiency×Sex | 1.00 | 0.90~1.12 | 0.939 |
| Sleep regularity index × Sex | 0.89 | 0.79~1.01 | 0.285 |
| Sleep midpoint×Sex | 0.99 | 0.88~1.12 | 0.939 |
| Relative amplitude×Sex | 0.94 | 0.86~1.02 | 0.285 |
| Sleep duration×Sleep efficiency | 0.98 | 0.95~1.02 | 0.317 |
| Sleep duration×relative amplitude | 0.96 | 0.93~0.99 | 0.012 |
| Sleep efficiency×relative amplitude | 0.98 | 0.95~1.02 | 0.360 |

HR, Hazard Ratio; CI, Confidence Interval.

**Table S5** **Objective sleep characteristics and subgroup analysis of osteoporosis**

|  | **HR** | **95% CI** | ***P*** |
| --- | --- | --- | --- |
| **Male** |  |  |  |
| Average sleep duration | 0.86 | 0.78~0.96 | 0.008 |
| Sleep efficiency | 0.93 | 0.83~1.03 | 0.169 |
| Sleep regularity index | 0.92 | 0.82~1.04 | 0.170 |
| Sleep midpoint | 1.00 | 0.89~1.12 | 0.990 |
| Relative amplitude | 0.77 | 0.69~0.85 | <0.001 |
| **Female** |  |  |  |
| Average sleep duration | 0.92 | 0.88~0.97 | 0.002 |
| Sleep efficiency | 0.93 | 0.88~0.97 | 0.002 |
| Sleep regularity index | 0.99 | 0.94~1.05 | 0.841 |
| Sleep midpoint | 1.03 | 0.98~1.09 | 0.255 |
| Relative amplitude | 0.83 | 0.79~0.88 | <0.001 |

HR, Hazard Ratio; CI, Confidence Interval.

**Table S6 Sensitivity analysis with 1-, 2-, and 3-year lag periods**

| **Characteristics** | **HR** | **95% CI** | ***P*** |
| --- | --- | --- | --- |
| Average sleep duration |  |  |  |
| 1-year lag | 0.93 | 0.88~0.98 | 0.004 |
| 2-year lag | 0.93 | 0.88~0.98 | 0.009 |
| 3-year lag | 0.93 | 0.88~0.99 | 0.018 |
| Sleep efficiency |  |  |  |
| 1-year lag | 0.93 | 0.88~0.98 | 0.004 |
| 2-year lag | 0.93 | 0.88~0.98 | 0.009 |
| 3-year lag | 0.92 | 0.87~0.98 | 0.005 |
| Sleep regularity index |  |  |  |
| 1-year lag | 1.00 | 0.95~1.06 | 0.937 |
| 2-year lag | 1.01 | 0.95~1.07 | 0.835 |
| 3-year lag | 1.02 | 0.96~1.09 | 0.542 |
| Sleep midpoint |  |  |  |
| 1-year lag | 1.01 | 0.96~1.06 | 0.759 |
| 2-year lag | 1.01 | 0.96~1.07 | 0.713 |
| 3-year lag | 1.03 | 0.97~1.09 | 0.365 |
| Relative amplitude |  |  |  |
| 1-year lag | 0.82 | 0.77~0.87 | <0.001 |
| 2-year lag | 0.82 | 0.77~0.87 | <0.001 |
| 3-year lag | 0.82 | 0.76~0.87 | <0.001 |

HR, Hazard Ratio; CI, Confidence Interval.

**Table S7 Sensitivity analysis using the Fine-Gray subdistribution hazard model**

| **Characteristics** | **HR** | **95% CI** | ***P*** |
| --- | --- | --- | --- |
| Average sleep duration | 0.92 | 0.87~0.96 | <0.001 |
| Sleep efficiency | 0.93 | 0.88~0.97 | 0.002 |
| Sleep regularity index | 0.98 | 0.93~1.03 | 0.430 |
| Sleep midpoint | 1.02 | 0.98~1.08 | 0.338 |
| Relative amplitude | 0.82 | 0.78~0.87 | <0.001 |

HR, Hazard Ratio; CI, Confidence Interval.

**Table S8 Sensitivity analysis censoring follow-up at December 31, 2019**

| **Characteristics** | **HR** | **95% CI** | ***P*** |
| --- | --- | --- | --- |
| Average sleep duration | 0.88 | 0.83~0.93 | <0.001 |
| Sleep efficiency | 0.90 | 0.85~0.96 | <0.001 |
| Sleep regularity index | 0.97 | 0.91~1.04 | 0.361 |
| Sleep midpoint | 1.03 | 0.96~1.09 | 0.404 |
| Relative amplitude | 0.79 | 0.74~0.85 | <0.001 |

HR, Hazard Ratio; CI, Confidence Interval.

**Table S9 Sensitivity analysis restricted to postmenopausal women**

| **Characteristics** | **HR** | **95% CI** | ***P*** |
| --- | --- | --- | --- |
| Average sleep duration | 0.93 | 0.88~0.98 | 0.021 |
| Sleep efficiency | 0.91 | 0.86~0.96 | 0.001 |
| Sleep regularity index | 1.01 | 0.95~1.08 | 0.734 |
| Sleep midpoint | 1.01 | 0.95~1.07 | 0.734 |
| Relative amplitude | 0.82 | 0.77~0.88 | <0.001 |

HR, Hazard Ratio; CI, Confidence Interval.

**Table S10 Sensitivity analysis excluding potential mediator-like variables**

| **Characteristics** | **HR** | **95% CI** | ***P*** |
| --- | --- | --- | --- |
| Average sleep duration | 0.90 | 0.86~0.95 | <0.001 |
| Sleep efficiency | 0.91 | 0.87~0.96 | <0.001 |
| Sleep regularity index | 0.93 | 0.88~0.98 | 0.005 |
| Sleep midpoint | 1.05 | 1.00~1.11 | 0.040 |
| Relative amplitude | 0.78 | 0.74~0.81 | <0.001 |

HR, Hazard Ratio; CI, Confidence Interval.

**Supplementary Method 1**

Raw triaxial accelerometer data were processed using the GGIR package (https://wadpac.github.io/GGIR/index.html). Briefly, raw signals were calibrated to local gravity, non-wear periods were detected, and acceleration was summarized as the Euclidean Norm Minus One (ENMO). Sleep periods were identified using the GGIR sleep detection algorithm, which detects sustained inactivity bouts based on changes in the arm angle and estimates the main sleep period across each 24-hour cycle. Average sleep duration was calculated as the mean sleep time across valid days, sleep efficiency as the proportion of the sleep period spent asleep, and sleep midpoint as the midpoint between sleep onset and wake time.

Relative amplitude was calculated as the difference between the average activity during the most active 10-hour period (M10) and the least active 5-hour period (L5), divided by their sum: (M10 − L5)/(M10 + L5). Sleep regularity index was calculated from the sleep–wake time series as the probability of being in the same sleep/wake state at any two time points 24 hours apart, scaled from −100 to 100, with higher values indicating more regular sleep–wake patterns.
